# Health related behaviors among HIV-infected people who are successfully linked to care: an institutional-based cross-sectional study

**DOI:** 10.1186/s40249-020-00642-1

**Published:** 2020-03-10

**Authors:** Jun-Fang Xu, Pei-Cheng Wang, Feng Cheng

**Affiliations:** 1 Center for Health Policy Studies, School of Public Health, Zhejiang University School of Medicine, Hangzhou, China; 2 Research Center for Public Health, School of Medicine, Tsinghua University, Beijing, China

**Keywords:** Health related behavior, HIV-infected people, Link to care, China

## Abstract

**Background:**

By the end of October 2019, there were 958 thousand people were reported living with HIV/AIDS in China. Unhealthy lifestyle factors, such as smoking, drinking alcohol, using illicit drugs and no physical activity have been found to mitigate the positive impact of antiretroviral therapy (ART) on viral load and HIV-related quality of life. Moreover, risky sexual behavior among HIV-positive persons places their partners at risk for HIV transmission and other sexually transmitted infections. The aim of the study is to determine the prevalence of unhealthy behavior of people living with HIV/AIDS and related influencing factors, particularly those that are closely connected with HIV infection and ART effects.

**Methods:**

An institutional based cross-sectional study design was used to collect data from people living with HIV/AIDS (PLWHA) in Beijing and Yunnan Province. The following information was included in the questionnaire survey: social-demographic characteristics, health behavior information, sexual risk behaviors. Binary logistic regression model was conducted to analyze the influencing factors of unhealthy general health behaviors and risky sexual behaviors.

**Results:**

In total, 2575 PLWHA were included in the study and 78.3% (2017/2575) were male. For the general health behaviors, 34.2% (987/2544) smoke; 33.8% (870/2575) drank alcohol and 2.3% (49/2134) reported the use of illicit drugs in the previous 6 months. From the sexual behaviors perspective, 59.0% (1519/2575) had sex in the previous 6 months. Among people who had sex, 92.0% (1398/1519) had fixed sexual partners. Among those with no fixed sexual partner, 38.0% (46/121) had more than three partners. Among men who had sex, 34.7% (448/1292) reported having sex with men in the previous 6 months and 16.7% (75/448) of these had group sexual activity. Among participants, 72.2% (1053/1458) used condoms every time they had sex while 6.4% (94/1458) of people never used condom. Male people living with HIV/AIDS were more likely to have sexual risk behaviors (adjusted odds ratio [*OR*] = 2.208, 95% confidence interval [*CI*]: 1.147–4.252) and unhealthy general health behaviors (adjusted *OR* = 2.029, 95% *CI*: 1.480–2.783). The odds of higher risk sexual behaviors was 1.546 times (95% *CI:* 1.302–1.827, *P* = 0.001) greater among participants who drank alcohol compared with their non-drinking counterparts.

**Conclusions:**

PLWHA is a group that is vulnerable to problematic health behaviors, especially for men who were more likely to drink alcohol, have more sexual partners, more sexual risk behaviors including group sexual activity, not using condoms and using drugs. Therefore, interventions focusing on gender-specific risk behaviors reduction for people living with HIV/AIDS are now necessary to control the spread of HIV infection and improve the efficacy of antiviral treatment.

## Background

By the end of 2018, there were 37.9 million people living with HIV/AIDS (PLWHA) globally and 1.7 million new HIV cases were diagnosed in that year [1, 2]. In China, about 958 thousand people were living with HIV/AIDS at the end of October 2019, and the number of HIV new infections is increasing from 60 thousand in 2008 to 140 thousand in 2018 [1, 3]. Moreover, with the introduction of free highly active antiroviral therapy (HAART) in 2003, the number of people receiving HAART increased from 12.6 thousand to 712 thousand in 2018 and the rates of death among HIV positive people decreased significantly in China [1, 3–5]. Correspondingly, the total number of people living with HIV/AIDS is increasing significantly, which means there are many long-term survivors with a long duration of infectiousness. Therefore, the importance of health behavior cannot be overstated among these people living with HIV/AIDS.

Research has shown that unhealthy lifestyles including smoking, lack of physical exercise and substance abuse put PLWHA at an increased rate of disease progression, opportunistic infections and chronic obstructive pulmonary diseases [6–9]. In additional, risky sexual behaviors among HIV positive people put their partners at risk for HIV infection and other sexually transmitted infections [10–12]. Moreover, the success of the “treatment as prevention” model also requires interventions that provide PLWHA the necessary skills to develop and sustain health-promoting behaviors including healthy sexual behaviors [13, 14]. Under the “Four Frees and One Care” policy in China, despite significantly increased access to ART treatment and continuous preventive efforts, there are still a large number of people being infected with HIV each year. Therefore, research focusing simultaneously on sexual risks and general health behaviors (e.g., alcohol/substance use) among HIV-infected people is critically needed. Currently there are few studies on such health risk behaviors among HIV-infected people who are successfully linked to care in China. Under this background, we aim to analyze the prevalence of unhealthy behaviors of people living with HIV/AIDS and related influencing factors, particularly those that are closely connected with HIV infection and ART effects.

## Methods

### Participants and data collection

We used an institutional based cross-sectional study design to collect data from HIV positive people who attended the AIDS clinics of hospitals located in Beijing between 24 May and 20 June, 2019, and those who received care in infectious disease hospitals and non-government organizations in Yunan Province (i.e., Kunming, Hekou, Lijiang, Xishuangbanna and Yuxi, which were cities with large number of PLWHA) between 5 April and 27 April, 2018. The inclusion criteria of participants were: 1) People who were diagnosed with HIV; 2) People who were more than 18 years old; 3) Successfully linked to HIV care and receiving antiretroviral therapy in the specialized hospitals or clinics; 4) Willing to participate in the study. The exclusion criteria were: 1) People who were under 18 years old; 2) Those who could not take care of themselves because of severe complications of HIV/AIDS or cognitive problems; 3) People who did not want to participant in the investigation. The questionnaire in our study included the following information: social-demographic characteristics (e.g., gender, age, marital status, educational level, employment, salary and living status), health behavior information (e.g., smoking, drinking alcohol, illicit drug use and physical exercise), sexual risk behaviors (type of sexual partners, number of sexual partners, condom use, homosexual and group sexual behaviors and illicit drug use). The questionnaire was paper-based structured questionnaire in Chinese. It was pre-tested and then revised on the basis of the pre-test.

Eligible people who expressed an interest in participating in the study to their attending physician were referred to an interviewer and the interviewer read the standardized informed consent form to them in the ART clinics. Participation was voluntary and the participants were informed that they could withdraw at any time. Possible inconveniences and advantages were also discussed with the participants on recruitment. The master students of public health, and volunteers in AIDS related non-government organizations were trained as interviewers before the investigation to help participants completing the questionnaires.

To maintain the confidentiality of participants’ information, the participants were allowed to sign their initials or use a pseudonym rather than real names on the consent form. After completing the informed consent procedure, the study participants were then administered to questionnaires in a face-to-face interview in a private setting. Unless the participants insisted on completing questionnaires without assistance, the interviewers read questions to them and then recorded their responses. After the survey, the interviewer then checked the completed hardcopy questionnaires for accuracy and completeness. Finally, 2575 people living with HIV/AIDS were incorporated in the study.

### Health behavior

General health behavior information including smoking, alcohol consumption and physical exercise habits was collected. Alcohol and tobacco use was assessed with the questions in supplementary table. According to Chinese drinking habits, the alcohol was divided into spirit, beer and grape wine in our questionnaire [15]. Fifty grams (Liang in Chinese), bottles (500 ml per bottle), goblets (180 ml per goblet) were used to measure the consumption of spirit, beer and grape wine respectively. Physical exercise was assessed by asking whether they did physical exercise or not. If they answered “yes” to this question, a follow-up question asked how often, what kind of exercise and how many minutes they did physical exercise for each time on average (See supplementary table).

### Sexual behavior

Sexual risk behavior data regarding the type of sexual partners, number of sexual partners, condom use, homosexual and group sexual behaviors and illicit drug using were collected from all participants. The specific questions are shown in the supplementary table.

To control the data quality, the interviewers were trained before the investigation; the physicians helped explain the aims of investigation to potential participants in order to avoid their considerations, which may partly benefit to the data authenticity. After the survey, the interviewer would then check the completed hardcopy questionnaires for accuracy and completeness and errors in questionnaire would be reviewed with participants. Before data analysis, logical check between different variables was performed in Statistical Analysis System 9.2(SAS Institute Inc., Cary, North Carolina, USA), including ranges, consistency and logical relationships. Whenever such checks revealed errors, original hard copy questionnaires were revisited for final confirmation and correction.

### Data analysis

The outcome variables were unhealthy general health behavior and sexual risk behavior. Unhealthy general health behavior was recognized if the people smoked, drank alcohol, used illicit drugs or took no physical exercise. Sexual risk behavior was defined as having one of the following sexual behaviors: more than one sexual partner, sex with same gender partners, sex with casual or one time partners, group sex or not using condoms during sex. Demographic data and health behavior was analyzed using descriptive statistics with frequency, percentage, mean and standard deviations. A binary logistic regression model was conducted to analyze the influencing factors of unhealthy general health behaviors and sexual risk behavior. Independent variables included: gender, age, marital status, living status, education level, salary, occupation, smoking, drinking alcohol and physical exercise. The statistical software SPSS 23.0 software (IBM, Armonk, NY, USA) was used to analyze all the data. Variables with *P* < 0.05 were considered as statistically significant.

## Results

### Basic characteristics

The basic characteristics of people living with HIV/AIDS were showed in Table 1. More than three quarters (78.3%, 2017/2575) were male; 82.8% (2071/2500) were younger than 50 years old; 45.8% (1173/2561) were unmarried, 36.5% (937/2568) had a bachelor degree or above. The salary of 73.5% people (1873/2548) was lower than RMB 5000 (USD 705.5). Among participants, 61.2% (1578/2575) lived with their family.

**Table 1:** The characteristics of people living with HIV/AIDS

Items	Number	%
**Gender**		
Male	2017	78.3
Female	558	21.7
**Age**		
18–29	562	22.5
30–39	878	35.1
40–49	631	25.2
50–59	273	10.9
≥ 60	156	6.2
**Marital status**		
Unmarried	1173	45.8
Married	1031	40.3
Divorced or widowed	348	13.6
Other	9	0.3
**Educational level**		
Junior high school and below	987	38.4
Senior high school	644	25.1
Undergraduate	860	33.5
Postgraduates and above	77	3.0
**Occupation**		
Employee	818	32.4
Students	62	2.4
Retired	149	5.9
Work part time	454	18.0
No work	454	18.0
Other	590	23.4
**Salary(RMB)**		
< 5000	1873	73.5
5000–10 000	421	16.5
≥ 10 000	254	10.0
**Living status**		
Alone	883	34.2
With family members	1578	61.2
Friends	79	3.1
Other	38	1.5

### General health behaviors

The status of general health behaviors among people living with HIV/AIDS were showed in Table 2. Among the participants, 38.8% (987/2544) smoked and 92.2% (910/987) of these were male; Among smokers, 76.1% (737/968) reported smoking ≥ eight cigarettes a day. About half of HIV positive people never do physical exercises. Among people doing physical exercise, 45.2% (602/1332) reported exercising regularly. Among participants, 33.8% (870/2575) of people living with HIV/AIDS drank alcohol and 90.4% (787/870) of these were male; the average alcohol consumption was 2.6 bottles, 132 g and 308.5 ml for beer, liquor and grape respectively.

**Table 2:** General health behaviors among people infected with HIV

Items	Number	%
**Smoking**		
Yes	987	38.8
No	1557	61.2
**Number of cigarettes per day**		
1	29	3.0
2–4	85	8.8
5–7	117	12.1
≥ 8	737	76.1
**Physical activity**		
Yes	1175	44.8
No	1448	55.2
**Frequency of exercise**		
Seldom	730	54.8
1–2 times/week	322	24.2
3–4 times/week	148	11.1
Almost each day	132	9.9
**Drinking alcohol**		
Yes	870	33.8
No	1705	66.2
**Alcohol consumption per time(M ± SD)**		
Beer (bottle)	2.6 ± 2.2	
Liquor (g)	132.0 ± 91.9	
Grape (ml)	308.5 ± 238.2	

Notes: “M” represents Mean value. “SD” is standard deviation. “g” is gram. “ml” is milliliter

### Sexual behaviors

**Fig. 1:**
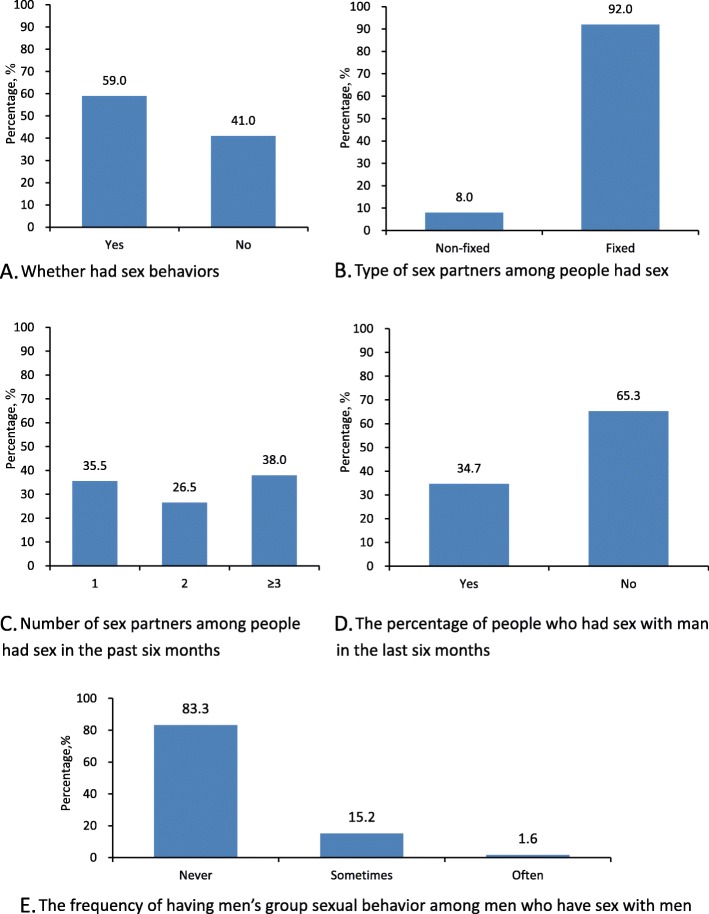
Sexual behaviors in previous 6 months

With regard to sexual behavior (Fig. 1), 59.0% (1519/2575) of participants reported having sex in the past 6 months, and 8.0% (121/1519) of the sex partners were not fixed. Among people who had sex with non-fixed partners, 64.5% (46/121) reported that the number of partners in the past 6 months was ≥2, and 97.8% (45/46) of these were men. Among men, 34.7% of men reported having sex with other men, and of these 16.7% had group sex.

**Fig. 2:**
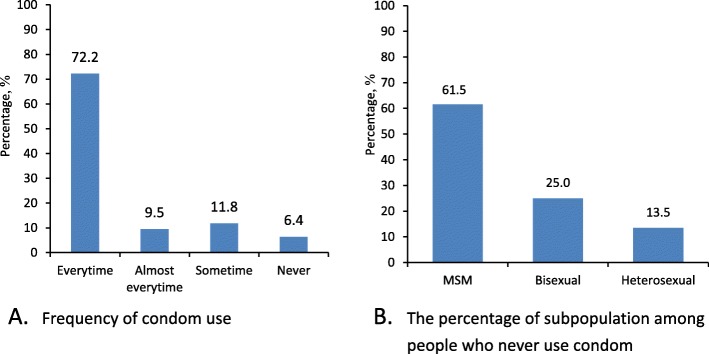
Condom use during sexual behaviors in the last 6 months

The condom using during sexual behaviors in the previous 6 months were presented in Fig. 2. Among people who had sex, 72.2% (1053/1458) of HIV positive people used a condom every time they had sex, and 6.4% (94/1458) said they never used condom. For people who never used condom, all were male including 61.5% men who have sex with men (MSM) and 25.0% bisexual male.

**Fig. 3:**
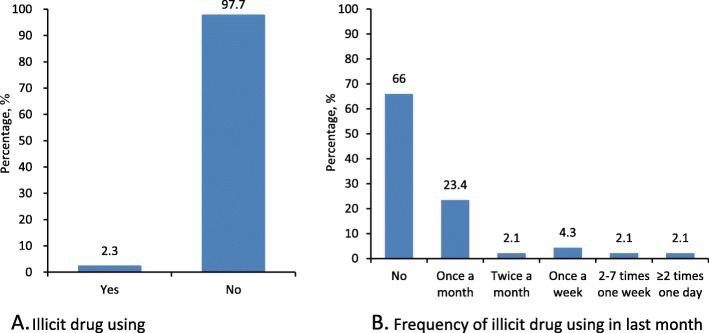
Illicit drug using among people living with HIV/AIDS

Illicit drug use among people with HIV/AIDS were illustrated n Fig. 3. Among participants, 2.3% (49/2134) admitted using illicit drugs in the previous 6 months, and 90.5% of them were men. Among illicit drug users, 23.4% used one time, 2.1% two times and 8.5% more than four times during the past month.

### Influencing factors

Binary logistic regression results are summarized in Table 3. Men with HIV were more likely to have sexual risk behavior (adjusted odds ratio [*OR*] = 2.208, 95% confidence interval [*CI*]: 1.147–4.252) and unhealthy general health behavior (adjusted *OR* = 2.029, 95% *CI*: 1.480–2.783). The odds of higher sexual risk behavior were 1.546 times (95% *CI:* 1.302–1.827, *P* = 0.001) greater for participants who had alcohol consumption habits compared with those who did not drink.

**Table 3:** The influencing factors of unhealthy general health behaviors and sexual risk behavior

Variables	Risk sexual behavior					Unhealthy general health behaviors				
	B	SE	*P*-value	Crude *OR* (95% *CI*)	Adjusted *OR* (95% *CI*)	B	SE	*P*-value	Crude *OR* (95% *CI*)	Adjusted *OR* (95% *CI*)
**Gender**										
Female (Reference)			.	1				.	1	
Male	0.792	0.3343	0.018	4.033 (3.151–5.163)	2.208 (1.147–4.252)	0.708	0.1611	< 0.001	1.947 (1.491–2.542)	2.029 (1.480–2.783)
**Age**										
≥ 60 (Reference)			.					.	1	
18–29	0.407	0.7355	0.580	13.582 (7.532–24.493)	1.503 (0.355–6.352)	0.659	0.3705	0.075	1.472 (0.876–2.472)	1.933 (0.935–3.996)
30–39	0.512	0.7204	0.477	8.169 (4.565–14.619)	1.669 (0.407–6.852)	0.770	0.3355	0.022	1.431 (0.875–2.339)	2.159 (1.119–4.167)
40–49	0.623	0.7060	0.378	3.857 (2.129–6.987)	1.864 (0.467–7.436)	0.556	0.3290	0.091	1.165 (0.706–1.921)	1.744 (0.915–3.324)
50–59	0.867	0.6927	0.211	3.333 (1.764–6.295)	2.379 (0.612–9.246)	0.892	0.3645	0.014	1.633 (0.889.3.001)	2.440 (1.194–4.986)
**Marital status**										
Divorced or widowed (Reference)			.	1				.	1	
Unmarried	−0.145	0.3035	0.633	3.139 (2.408–4.093)	0.865 (0.477–1.568)	−0.493	0.2652	0.063	0.844 (0.555–1.283)	.611 (0.363–1.027)
Married	−0.385	0.2946	0.191	0.724 (0.545–0.961)	0.680 (0.382–1.212)	−0.354	0.2364	0.134	0.648 (0.427–0.982)	.702 (0.442–1.115)
**Living status**										
Friends (Reference)			.	1				.	1	
Alone	−0.207	0.3127	0.508	0.644 (0.439–0.945)	0.813 (0.440–1.501)	0.437	0.3341	0.191	1.276 (0.686–2.373)	1.548 (0.804–2.979)
With family members	−0.357	0.3243	0.271	0.324 (0.222–0.472)	0.700 (0.371–1.322)	0.157	0.3313	0.636	0.88 (0.485–1.596)	1.170 (0.611–2.240)
**Education level**										
Postgraduates and above (Reference)			.	1				.	1	
Junior high school and below	0.013	0.4593	0.978	0.402 (0.250–0.646)	1.013 (0.412–2.492)	0.171	0.4246	0.688	0.849 (0.414–1.743)	1.186 (0.516–2.726)
Senior high school	0.180	0.4078	0.659	0.768 (0.476–1.239)	1.197 (0.538–2.663)	0.585	0.4178	0.161	1.266 (0.602–2.661)	1.795 (0.792–4.072)
Undergraduate	0.284	0.3778	0.452	1.283 (0.801–2.053)	1.328 (0.633–2.786)	0.501	0.3893	0.198	1.301 (0.626–2.704)	1.651 (0.770–3.541)
**Salary**										
≥ 10 000 (Reference)			.	1				.	1	
< 5000	0.305	0.2584	0.238	0.893 (0.681–1.172)	1.356 (0.817–2.250)	0.44	0.2608	0.092	1.179 (0.792–1.755)	1.552 (0.931–2.588)
5000–10 000	0.198	0.2234	0.376	1.339 (0.975–1.839)	1.219 (0.787–1.888)	0.142	0.2732	0.604	1.164 (0.721–1.878)	1.152 (0.674–1.968)
**Occupation**										
No work (Reference)			.	1				.	1	
Employee	−0.540	0.2527	0.033	1.814 (1.457–2.258)	0.583 (0.355–0.956)	−0.14	0.2243	0.533	1.098 (0.779–1.547)	.869 (0.560–1.349)
Students	−1.866	0.7243	0.010	2.141 (1.274–3.599)	0.155 (0.037–0.640)	0.356	0.5815	0.541	1.822 (0.641–5.179)	1.427 (0.457–4.461)
Retired	−0.612	0.6638	0.356	0.388 (0.241–0.624)	0.542 (0.148–1.992)	0.525	0.4067	0.197	1.397 (0.734–2.659)	1.691 (0.762–3.752)
Work part time	−0.319	0.2711	0.239	1.003 (0.805–1.249)	0.727 (0.427–1.236)	−0.265	0.1765	0.134	0.834 (0.606–1.15)	.768 (0.543–1.085)
**Smoking**										
No (Reference)			.	1						
Yes	−0.182	0.1855	0.325	0.975 (0.827–1.151)	0.833 (0.579–1.199)					
**Drinking alcohol**										
No (Reference)			.	1						
Yes	0.608	0.1753	0.001	1.546 (1.308–1.827)	1.836 (1.302–2.588)					
**Physical exercise**										
No (Reference)			.	1						
Yes	0.034	0.1658	0.836	0.988 (0.750–1.301)	1.035 (0.748–1.432)					

B: regression coefficient; *SE* standard error, *OR* odds ratio, *CI* confidence interval

## Discussion

Our results show that health risk behaviors, including sexual risk behavior, exist among HIV-positive persons who are successfully linked to care and ART treatment in China. For the general health behaviors, 33.8% of PLWHA drank alcohol, which was slightly higher than earlier research done in other countries, which showed approximately 25% used alcohol in Africa and 21.8% in the United States in previous month [16, 17]. Evidence has shown that the health outcomes are worse in patients who often drink alcohol, including a higher incidence of psychosocial problems and lower treatment adherence levels [18–22]. In addition, most of these drinkers were male PLWHA, which was consistent with findings from other studies [23–25].

From the perspective of sexual behavior, 59.0% of the participants had sex in the last 6 months, and 72.2% of these reported using a condom every time when they had sex. This is consistent with studies conducted in Jamaica [26]. Although a high percent of HIV-positive individuals use condoms, condom use is still far from universal among HIV-infected persons. For example, a considerable number of HIV-positive persons (6.4%) never used condom in our study, which put others at risk for infection and places themselves at risk for re-infection with new HIV strains or other sexual transmitted diseases [27]. The percentage of participants using illicit drugs among PLWHA was 2.3% in our study. However, this figure may much lower than the real figure because this study was based on the self-reported data from HIV positive people who may tend not to admit to using drugs.

Moreover, the odds of higher risk sexual behavior was 1.546 times (95% *CI:* 1.302–1.827; *P* = 0.001) greater for participants who drink alcohol compared with those who do not drink. This may explain why the sexual risks are higher for men, who tend to drink more, than for women. Indeed, men do have a higher sexual risk than women, as they are more likely to have more than one partner, to take sexual risks including never using condom and use drugs more. It was also statistically significant that men living with HIV were more likely to take part in risky sexual behavior (adjusted *OR* = 2.208, 95% *CI*: 1.147–4.252) and unhealthy general health behavior (adjusted *OR* = 2.029, 95% *CI:* 1.480–2.783). Thus, there is an urgent need to develop behavioral interventions to promote safe sex practices among HIV-positive individuals, especially male PLWHA.

It is a universally accepted truth that behavior intervention is the key to prevent and control HIV [28–30]. Behavioral intervention in China including individual consultation, health education (including peer education), free condom distribution, regular HIV testing has been carried out for years [31, 32]. However, these traditional face-to-face intervention methods are time-consuming and laborious, with high costs and limited audience [31]. Moreover, the effects of these interventions are decreasing.

It is vitally important to explore more economical, accessible and effective intervention methods, particularly for men with HIV/AIDS related problems. Providing more targeted health education, consultation on healthy lifestyles, and conducting motivational interviews are ideas that could help reduce the incidence of high-risk behavior in key populations.

This study is subject to some limitations. Firstly, all participants were recruited from health care facilities in Beijing and Yunnan Province so the results may not be representative for all PLWHA in China. Secondly, the data was all self-reported, so some recall and social desirability bias is inevitable. For example, some participants may not provide an honest response on sexual risk behavior or illicit drug using, which are not socially desirable. This may underestimate the prevalence of unhealthy behaviors of people living with HIV/AIDS.

## Conclusions

PLWHA who were successfully linked with care are a group that is vulnerable to problematic health behaviors, especially for men, who are more likely to drink alcohol, have more sex partners, have more risky sexual behavior including group sex, without condom and using drugs. Therefore, interventions focusing on gender-specific risk behavior reduction for people living with HIV/AIDS are now essential to control the spread of HIV infection and improve the efficacy of antiviral medication.

## Supplementary information

**Additional file 1 Table S1.** The questions and assessment regarding the health behaviors of people living with HIV/AIDS.

## Data Availability

All of the main data have been included in the results. Additional materials with details may be obtained from the corresponding author.
