# Supplementary material for: Health related behaviors among HIV-infected people who are successfully linked to care: an institutional-based cross-sectional study
Source: Infect Dis Poverty. 2020 Mar 10;9:28. doi: 10.1186/s40249-020-00642-1 (PMC7068930; doi:10.1186/s40249-020-00642-1)
Supplement: Supplementary file 1 — Additional file 1 Table S1. The questions and assessment regarding the health behaviors of people living with HIV/AIDS. [file 40249_2020_642_MOESM1_ESM.docx]

# Table S1. The questions and assessment regarding the health behaviors of people living with HIV/AIDS

| **Questions** | **Assessment** |
| --- | --- |
| Do you currently drinking alcohol? | Yes/No |
| If “yes”, how much alcohol do you drink on average per time | Actual value |
| Do you currently smoking? | Yes/No |
| If “yes”, how many cigarettes do you smoke on average per day? | Actual value |
| Have you used illicit drugs in the past 6 month? | Yes/No |
| If “yes”, how often do you use illicit drugs? | Once a month/Twice a month/once a week/2-7 times one week/≥2 times one day |
| Do you do physical exercise？ | Yes/No |
| If “yes”, how often do you do physical exercise each time on average? | Seldom/1-2times one week/3-4times one week/Almost each day |
| If “yes”, how many minutes do you do physical exercise each time on average? | Actual value |
| Have you had sex in the past 6 month？ | Yes/No |
| How many sex partners do you have in last 6 month？ | Actual value |
| Have you had sex with same gender partners？ | Yes/No |
| Have you had sex with casual or one –time partners？ | Yes/No |
| Have you had group sex behaviors? | Yes/No |
| If “yes”, how often do you have group sex behaviors? | Never/Sometimes/Often |
| How often do you use condom in the sexual intercourse？ | Everytime/Almost everytime/Sometime/Never |
